# Cochlear coverage with lateral wall cochlear implant electrode arrays affects post-operative speech recognition

**DOI:** 10.1371/journal.pone.0287450

**Published:** 2023-07-12

**Authors:** Tobias Weller, Max Eike Timm, Thomas Lenarz, Andreas Büchner

**Affiliations:** 1 Department of Otorhinolaryngology, Hannover Medical School, Hannover, Germany; 2 German Hearing Center (DHZ), Hannover, Germany; 3 Cluster of Excellence “Hearing4All”, Oldenburg, Germany; Universidade Federal de Sao Paulo/Escola Paulista de Medicina (Unifesp/epm), BRAZIL

## Abstract

**Objectives:**

The goal was to investigate the relationship between the insertion angle/cochlear coverage of cochlear implant electrode arrays and post-operative speech recognition scores in a large cohort of patients implanted with lateral wall electrode arrays.

**Methods:**

Pre- and post-operative cone beam computed tomography scans of 154 ears implanted with lateral wall electrode arrays were evaluated. Traces of lateral wall and electrode arrays were combined into a virtual reconstruction of the implanted cochlea. This reconstruction was used to measure insertion angles and proportional cochlear coverage. Word recognition scores and sentence recognition scores measured 12 months after implantation using electric-only stimulation were used to examine the relationship between cochlear coverage/insertion angle and implantation outcomes.

**Results:**

Post-operative word recognition scores and the difference between post- and pre-operative word recognition scores were positively correlated with both cochlear coverage and insertion angle, however sentence recognition scores were not. A group-wise comparison of word recognition scores revealed that patients with cochlear coverage below 70% performed significantly worse than patients with coverage between 79%-82% (p = 0.003). Performance of patients with coverage above 82% was on average poorer than between 79%-82, although this finding was not statistically significant (p = 0.84). Dividing the cohort into groups based on insertion angle quadrants revealed that word recognition scores were highest above 450° insertion angle, sentence recognition scores were highest between 450° and 630° and the difference between pre- and post-operative word recognition scores was largest between 540° and 630°, however none of these differences reached statistical significance.

**Conclusions:**

The results of this study show that cochlear coverage has an effect on post-operative word recognition abilities and the benefit patients receive from their implant. Generally, higher coverage led to better outcomes, however there were results indicating that insertion past 82% cochlear coverage may not provide an additional benefit for word recognition. These findings can be useful for choosing the optimal electrode array and thereby improving cochlear implantation outcomes on a patient-individual basis.

## Introduction

Since their first commercial use about 40 years ago, cochlear implants (CIs) have become the preferred treatment option for patients with severe to profound hearing loss, and, in more recent years, even for patients with a substantial amount of residual hearing at low- to mid-frequencies. Notwithstanding the overwhelming general success of CIs, outcomes remain highly variable and can differ considerably between patients [1]. Numerous studies have investigated which ones of the many factors that play a role in the treatment with a CI have the strongest effect on treatment success and the level of post-operative hearing performance that CI patients achieve. These factors include, but are not limited to, duration of deafness, age at implantation, etiology of the hearing loss, duration of hearing aid use prior to surgery, and pre-operative speech recognition [2–7]. Apart from these pre-operatively determined demographic and audiometric factors, research has focused on the impact of surgical factors such as the surgical approach (cochleostomy versus round window; [8, 9]), the scalar position of the electrode array (scala tympani versus scala vestibuli; [9, 10]), and electrode array malposition such as a tip fold-over [11]. Furthermore, the influence of electrode array insertion depth and angle on speech recognition has been a heavily studied subject [12–25]. These parameters are of particular clinical interest because they are comparatively easy to manipulate by selecting electrode arrays of different type and/or length.

Dorman et al. [12] investigated the potential interaction between insertion depth and implantation outcomes in a study with normal hearing subjects. They simulated CIs with five electrodes using vocoded signals, and varied the simulated insertion depth between 22mm– 25mm by shifting the presented speech material to different vocoder frequencies. In all four tests that were conducted, their subjects showed better results for deeper simulated insertion depths. Hochmair et al. [13] simulated different insertion depths of lateral wall electrode arrays in a study with CI subjects by de-activating the four most apical electrode contacts while keeping the full input frequency range. With this reduced insertion depth, subjects performed significantly worse than with all twelve electrode contacts activated in both monosyllabic word recognition as well as sentence recognition in noise. In a condition with the same number of de-activated electrode contacts evenly distributed across the electrode array, subjects reached the same performance as with all electrode contacts activated, suggesting that hearing performance would be affected by insertion depth rather than the number of inactive electrode contacts. In a prospective trial, Buchman et al. [14] compared post-operative outcomes between two groups of CI subjects implanted with different lateral electrode arrays. One group of subjects was implanted with an electrode array with a length of 31 mm, while the other group of subjects was implanted with a shorter electrode array with a length of 24 mm but the same number of electrode contacts as the longer array. They measured consonant-nucleus-consonant (CNC) word recognition and sentence recognition in quiet and noise at multiple time points up to 12 months after the surgery and observed significantly better results in all tests in the group with the longer electrode array. These results caused the institutional review board to terminate the study because it no longer deemed it ethical to implant patients with the shorter electrode array. In a follow-up study, Canfarotta et al. [15] later published long-term results of those patients that had been enrolled in the study before its discontinuation. Even 48 months after surgery, patients with the longer electrode array performed better than those with the shorter electrode array, suggesting that the initial disadvantage introduced by the shorter electrode array could not be made up for by a longer time to adapt. In another retrospective study, Büchner et al. [16] also compared post-operative outcomes between three groups of patients with lateral wall electrode arrays of different length (20mm, 24mm, and 28mm). When looking at monosyllabic word recognition and sentence recognition in quiet and in noise they found significantly better results in the group with the longest electrode array at three months after activation. At six months after activation, patients with longer electrode arrays still showed better performance than those with shorter electrode arrays but the results did not reach statistical significance.

Using electrode array length as a parameter when analyzing CI outcomes serves as a good approximation, however, it does not take into account neither fluctuations in the surgically realized insertion depth nor individual differences of the inner ear morphology. If adequate imaging data is available, measuring insertion angle (IA) and/or cochlear coverage (CC), i.e. the proportion of the cochlear duct length that is covered by the electrode array, should result in a more precise assessment. Consequently, several studies in the past have used some form of radiological imaging data to investigate the effect of different surgical parameters on implantation outcomes. Skinner et al. [17] used computed tomography (CT) scans to determine cochlear length and electrode array insertion depth in a cohort of 26 patients with lateral wall electrode arrays. After calculating the CC, they correlated their results with word recognition scores obtained at least 12 months after surgery and found a positive linear correlation between those word recognition scores and CC. Yukawa et al. [18] used radiographs to determine the IA in a cohort of 48 patients with lateral wall electrode arrays and found a positive linear correlation between measured IAs and both phoneme recognition scores in quiet and sentence recognition scores in noise. O’Connell et al. [19, 20] measured IAs using CT scans and found a positive linear correlation between IA and word recognition scores both in a larger cohort with mixed electrode array types (N = 137) as well as in a smaller cohort with lateral wall electrode arrays (N = 48). For the larger cohort they also tested for a correlation between IA and sentence recognition scores but did not find statistically significant results. In a cohort of 54 patients with lateral wall electrode arrays Helbig et al. [21] found a positive linear correlation between IA and monosyllabic word recognition scores at twelve months after implantation. Contrary to these findings, van der Marel et al. [22] could not find any significant correlation between post-operative word and phoneme recognition scores and 6 different electrode position parameters (including IA and insertion depth) derived from multiplanar CT scans in a cohort of 203 patients with lateral wall electrode arrays. Lo Russo et al. [23] divided a cohort of 50 patients implanted with different types of electrode arrays into two groups based on the difference between post- and pre-operative disyllabic word recognition scores (ΔSDS). They analyzed flat-panel CT scans and found that the surgical insertion depth (SID, distance between basal electrode contact and round window) was significantly higher in the group with higher ΔSDS. However, they did not find significant differences in neither linear insertion depth nor IA between the two groups.

More recently, Cooperman et al. [24] measured the cochlear duct length (CDL) in CT scans of a cohort of 61 patients implanted with lateral wall electrode arrays using specialized software. They then estimated the proportional coverage of the cochlear duct using the electrode array length provided by the manufacturer (either 24mm, 28mm, or 31.5mm). When analyzing the effect of this coverage on CI performance, the results suggested that speech recognition was poorer for both very shallow and very deep insertions with an optimum in between. More generally, they concluded that cochlear coverage was more closely related to speech recognition scores than electrode array length alone. In a similar study with a cohort of 75 patients implanted with lateral wall electrode arrays, Canfarotta et al. [25] found a significant positive correlation between IA and word recognition scores, however they also observed that IAs above 600° did not provide any further benefit.

Even though some studies did not find a correlation between CI outcomes and positional parameters, overall previous results seem to suggest that the position of the electrode array in the cochlea generally plays a role in post-operative performance with a CI. Furthermore, this effect may be better captured when individual differences in insertion depth and cochlear length are taken into account. Several studies have clearly shown a correlation between IA and post-operative phoneme or word recognition scores, while results for a correlation between IA and sentence recognition scores have sometimes been less conclusive [20]. Some evidence has been published that the surgical insertion depth (i.e. the insertion depth of the most basal electrode contact) could also have an effect on performance with a CI.

However, most of these studies have investigated either small cohorts, or cohorts including different electrode array designs, different surgical approaches, or cohorts with varying degrees of scalar dislocation. The majority of these studies also evaluated IA as the main parameter for insertion depth, not taking into account individual differences in cochlear length. This study therefore aims at investigating potential effects of both insertion angle as well as cochlear coverage on post-operative speech perception with a CI in a large cohort that is homogenous in electrode array design and surgical approach. This is of particular clinical relevance because insertion angle and cochlear coverage can be planned (to some extent) before surgery. Consequently, if an ideal range of CC could be identified, speech recognition ability with the CI could be optimized on a patient-individual basis by customizing the choice of electrode array during pre-operative planning.

## Materials and methods

### Ethics statement

The Ethics committee of the Medical University of Hannover, Germany, approved this retrospective study. Patients gave written informed consent to the retrospective analysis of their data before their admission to the clinic. All patient data were anonymized prior to the retrospective analysis.

### Subjects

Subjects were selected from the database of CI patients at the German Hearing Center (DHZ) in Hanover, Germany. Patients were included in the cohort if they had been at least 18 years old at the time of implantation, had acquired their hearing impairment post-lingually, were German speaking, and if both post-operative speech recognition data as well as pre- and post-operative cone beam computed tomography (CBCT) imaging data were available. Patients were excluded from the cohort if they had been deaf for more than ten years prior to implantation with a CI (self-reported duration of deafness), had been diagnosed with vestibular schwannoma, or were fitted with electric-acoustic stimulation at the time of their post-operative speech recognition tests. Furthermore, patients were excluded if they suffered from mental, cognitive, or other conditions that prevented the patients from performing the speech recognition tests according to the protocol. One patient was excluded due to stimulation of the facial nerve on almost all electrodes, which led to a fitting with heavily reduced M-levels. If patients were implanted bilaterally, both ears were generally included. However, only the first implant a patient had received on either side was considered (i.e. no re-implantations). Additionally, individual ears were also excluded from the cohort if the first implant had been revised (i.e. the patient underwent a second surgery on the ear without re-implantation) or the ear had been re-implanted within the first two years after implantation, suggesting technical or medical issues that interfered with a regular adaptation process and learning curve. In an effort to reduce the impact of confounding factors concerning electrode array design, only ears implanted with a lateral wall electrode array of the FLEX family (MED-EL, Austria) were considered for this study.

### Imaging data analysis

All CBCT imaging data were analyzed using the DICOM viewer Osirix MD (v.7.0.2, Pixmeo, Switzerland). This software enables the user to trace structures in volumetric datasets and export the resulting traces. Using this feature, the lateral wall of the cochlea was traced in the pre-operative imaging datasets, starting at the round window and stopping at the apex of the cochlea. In the post-operative imaging datasets, the electrode array was traced by first putting a marker in the round window and then putting a marker on each intracochlear electrode contact. A more detailed description of this approach including a tutorial video can be found in [26].

All tracings were conducted by a trained researcher. First, tracings in both pre- and post-operative scans were done for all patients in the cohort, then those traces were visually checked and, if necessary, corrected in a second round of assessment by the same researcher.

**Fig 1:**
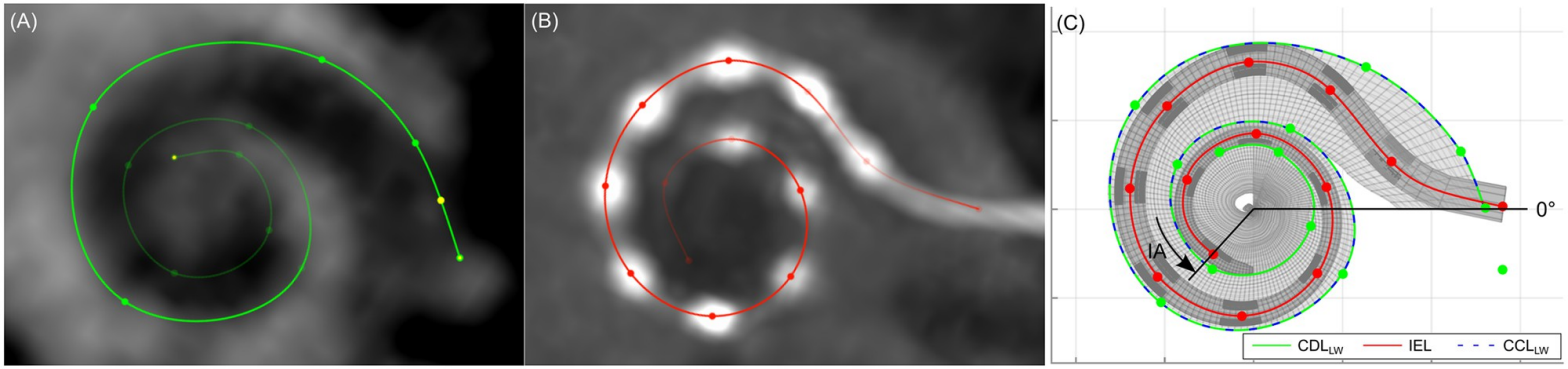
Visualization of the imaging data analysis method. First, pre-operative CBCT scans are used to trace the lateral wall of the cochlea (A). Then, post-operative CBCT scans are used to trace the electrode array by marking the position of each electrode contact (B). Finally, the two traces are combined to obtain a reconstruction of the implanted cochlea that can be used to derive anatomical and positional parameters (C).

The obtained traces were exported and further processed using Matlab (version 2018a, MathWorks, USA). First, the traced electrode array of each patient was registered onto the corresponding trace of the lateral wall using the method proposed by Schurzig et al. [27], resulting in a virtual reconstruction of the implanted cochlea. Then, the reconstructed cochlea was evaluated with respect to the cochlear duct length (CDL, length of the lateral wall from the center of the round window to the apex), the insertion angle (IA, angle between the center of the round window, the modiolus, and the most apical electrode contact), the covered cochlear length (CCL, length of the lateral wall from the center of the round window to the IA), and the inserted electrode length (IEL, length of intracochlear part of the electrode array from the round window to the apical electrode contact). A visualization of the imaging data evaluation process is shown in Fig 1. Finally, the cochlear coverage (CC) was computed as the proportion of the lateral wall covered by the electrode array:

$$CC=\frac{CCL}{CDL}$$

### Clinical data

Pre-operatively, speech recognition was assessed using the Freiburger Monosyllabic Word Test (FMWT; [28, 29]). A list of 20 words was presented monaurally over headphones at sound levels of 60 dB SPL, 80 dB SPL, 100 dB SPL, and, if tolerated by the subject, at 110 dB SPL to determine the maximum speech recognition at the optimal sound level (dB_opt_).

Post-operative speech recognition was assessed at 12 months after the initial fitting of the device using two different tests: The FMWT presented in quiet at a sound level of 65 dB SPL, and the German language Hochmair-Desoyer, Schulz, Moser (HSM) sentence test [30] presented in steady-state, speech-shaped noise at a signal-to-noise ratio (SNR) of 10 dB, with the sentences presented at a sound level of 65 dB SPL. Both tests were conducted within the general clinical routine, presented from a loudspeaker at a distance of 1 m in front of the subject. Hearing aids or CIs on the contralateral side were switched off during testing and the contralateral ear was plugged if contralateral hearing thresholds were lower than 70 dB HL at at least one frequency between 250 Hz and 3000 Hz. Additionally, the contralateral ear was masked with speech-shaped noise if hearing thresholds were lower than 30 dB HL at at least one frequency between 250 Hz and 3000 Hz, which was the case for 14 subjects. For both tests, scores are the percentage of words correctly repeated by the subject. The improvement in speech recognition due to treatment with a CI was computed as the simple difference between post- and pre-operative FMWT scores (FMWTpost—FMWT_pre_) and is denoted ΔFMWT.

### Statistical analysis

All statistical analyses were carried out using Python (version 3.8.8, Python Software foundation, USA) using the packages scipy (version 1.6.2), statsmodels (version 0.12.2), sklearn (version 0.23.2), and scikit_posthocs (version 0.6.7).

## Results

### Cohort

The subject selection process resulted in a cohort of 142 subjects that had been implanted at the Medical School Hanover in the time between 2009 and 2020. All subjects had their CI fitted by experienced CI audiologists at the DHZ. Subjects were predominantly female (N = 85, 59.9%) and the mean and median age at implantation was 63.5 years and 65.2 years, respectively (range: 19–88.6 years). The mean and median duration of hearing impairment before treatment with a CI (as reported by the patients) was 24.9 years and 22 years respectively (range: 0.5–69.1 years). Twelve of the subjects were implanted bilaterally with FLEX electrode arrays and both ears were included in the cohort, yielding a total of 154 ears to be analyzed. Of those, 13 ears were implanted with a FLEX20 electrode array (8.4%), 23 were implanted with a FLEX24 electrode array (15%), 2 were implanted with a FLEX26 electrode array (1.3%), 115 were implanted with a FLEX28 electrode array (74.7%), and 1 was implanted with a FLEXSOFT electrode array (0.65%). There were five ears with incomplete insertion: One FLEX20 (1 extracochlear electrode), one FLEX24 (2 extracochlear electrodes), and three FLEX28 (one with 2 extracochlear electrodes and two with 1 extraochlear electrode), and all of those extracochlear contacts were also disabled. All of the incomplete insertions were due to the surgeon experiencing resistance during the insertion of the electrode array and consequently abstaining from the application of a stronger insertion force in order to prevent trauma to the inner ear. Additionally, there were 17 ears where the electrode array was inserted completely, but one or more electrode contacts were disabled. Of these 17 ears, 15 ears had one contact disabled, and two ears had two contacts disabled. In one of these 17 ears, contact 6 in the middle of the electrode array was disabled, in all other cases the one or two most basal contacts were disabled.

The FLEX28 electrode array was used as the standard of care for patients with electric-only stimulation. Generally, patients with shorter electrode arrays had originally been scheduled for electric-acoustic stimulation (EAS), but either had no usable residual hearing after surgery (i.e. no objective or subjective benefit of the acoustic component at the initial fitting of the device), did not want to wear the acoustic component or could not wear it due to medical reasons, and were thus fitted with full electric stimulation. Cochlear length measurements were done preoperatively in some cases from 2018, but there was no guideline for their application in choosing a particular electrode array. Ultimately, electrode array selection remained at the surgeon’s discretion. All ears were implanted using the round-window approach. An overview over the demographics of the cohort is given in Table 1.

**Table 1:** Overview of the demographic data, anatomic measurement data and speech recognition data of the study cohort in groups according to electrode array.

**Electrode array**	**N**	**Demographic data**			
		**Duration of hearing impairment [yrs]**	**Age at implantation [yrs]**	**Pre-operative low-frequency PTA [dB HL]**	**Post-operative low-frequency PTA [dB HL]**
FLEX20	13	23.4 ± 17.4	58.8 ± 18.4	61.4 ± 17.0	87.0 ± 15.1
	(8.4%)	(3.4–69.0)	(19.4–75.0)	(38.3–96.7)	(58.3–103.3)
FLEX24	23	25.1 ± 13.7	65.0 ± 12.9	67.8 ± 19.7	88.1 ± 14.2 (N = 22)
	(14.9%)	(4.4–59.1)	(28.9–78.9)	(26.7–110.0)	(58.3–110.0)
FLEX26	2	21.3 ± 7.9	67.1 ± 1.2	79.2 ± 22.4	97.5 ± 13.0
	(1.3%)	(15.7–26.9)	(66.3–67.9)	(63.3–95.0)	(88.3–106.7)
FLEX28	115	25.1 ± 15.7	63.0 ± 13.7	76.3 ± 17.0	97.1 ± 11.9 (N = 109)
	(74.7%)	(0.5–66.5)	(19.0–88.6)	(25.0–106.7)	(63.3–110.0)
FLEXSOFT	1	23.9	77.3	66.7	106.7
	(0.65%)				
		**Anatomic measurement data**			
		**One-turn length (1TL) [mm]**	**Two-turn length (2TL) [mm]**	**Insertion angle (IA) [°]**	**Cochlear coverage (CC) [%]**
FLEX20	13	22.6 ± 1.0	35.1 ± 1.7	341 ± 29	56.6 ± 5.2
	(8.4%)	(20.4–24.2)	(31.7–37.3)	(281–383)	(50.6–70.0)
FLEX24	23	22.9 ± 0.9	35.4 ± 1.4	447 ± 42	67.6 ± 5.8
	(14.9%)	(20.8–24.4)	(32.7–38.1)	(315–512)	(51.8–79.9)
FLEX26	2	22.3 ± 1.1	34.8 ± 1.8	531 ± 26	75.5 ± 1.1
	(1.3%)	(21.6–23.1)	(33.5–36.2)	(512–549)	(74.7–76.3)
FLEX28	115	23.1 ± 1.0	35.8 ± 1.6	559 ± 49	78.8 ± 4.7
	(74.7%)	(20.2–25.2)	(31.8–39.7)	(421–678)	(66.2–91.2)
FLEXSOFT	1	23.6	36.9	580	80.1
	(0.65%)				
		**Speech recognition data**			
		**Pre-operative FMWT [%-correct]**	**Post-operative FMWT [%-correct]**	**HSM sentence test in noise [%-correct]**	**ΔFMWT [%-points]**
FLEX20	13	30.0 ± 26.2 (N = 12)	45.0 ± 26.1	38.2 ± 25.0	17.1 ± 28.1 (N = 12)
	(8.4%)	(0–75)	(10–90)	(0.0–75.5)	(-40.0–65.0)
FLEX24	23	29.5 ± 25.5 (N = 21)	50.0 ± 23.5	43.6 ± 28.0 (N = 20)	21.9 ± 23.6 (N = 21)
	(14.9%)	(0.0–65.0)	(5.0–95.0)	(0.0–89.6)	(-15.0–75.0)
FLEX26	2	35.0 ± 35.3	70.0 ± 0.0	69.4 ± 11.4	35.0 ± 35.3
	(1.3%)	(10.0–60.0)	(70.0–70.0)	(61.3–77.4)	(10.0–60.0)
FLEX28	115	24.0 ± 21.8 (N = 90)	59.4 ± 20.5	48.7 ± 26.4 (N = 107)	36.5 (N = 90)
	(74.7%)	(0.0–90.0)	(5.0–100.0)	(0.0–97.2)	(-25.0–85.0)
FLEXSOFT	1	-	85.0	34.9	-
	(0.65%)				

Demographic data, anatomic measurement data, and speech recognition data of the study cohort across the different electrode arrays. Given are the mean values ± one standard deviation and below in brackets the minimum and maximum values. Low-frequency pure tone thresholds (PTA) were determined as the mean thresholds across 125 Hz, 250 Hz, and 500 Hz. Pre-operative pure-tone threshold values are taken from the last measurement before implantation, post-operative thresholds are taken from the measurement at the time of the initial fitting of the implant. In the case of missing values, the remaining sample size is given in brackets.

### Cochlear size and electrode array placement

**Fig 2:**
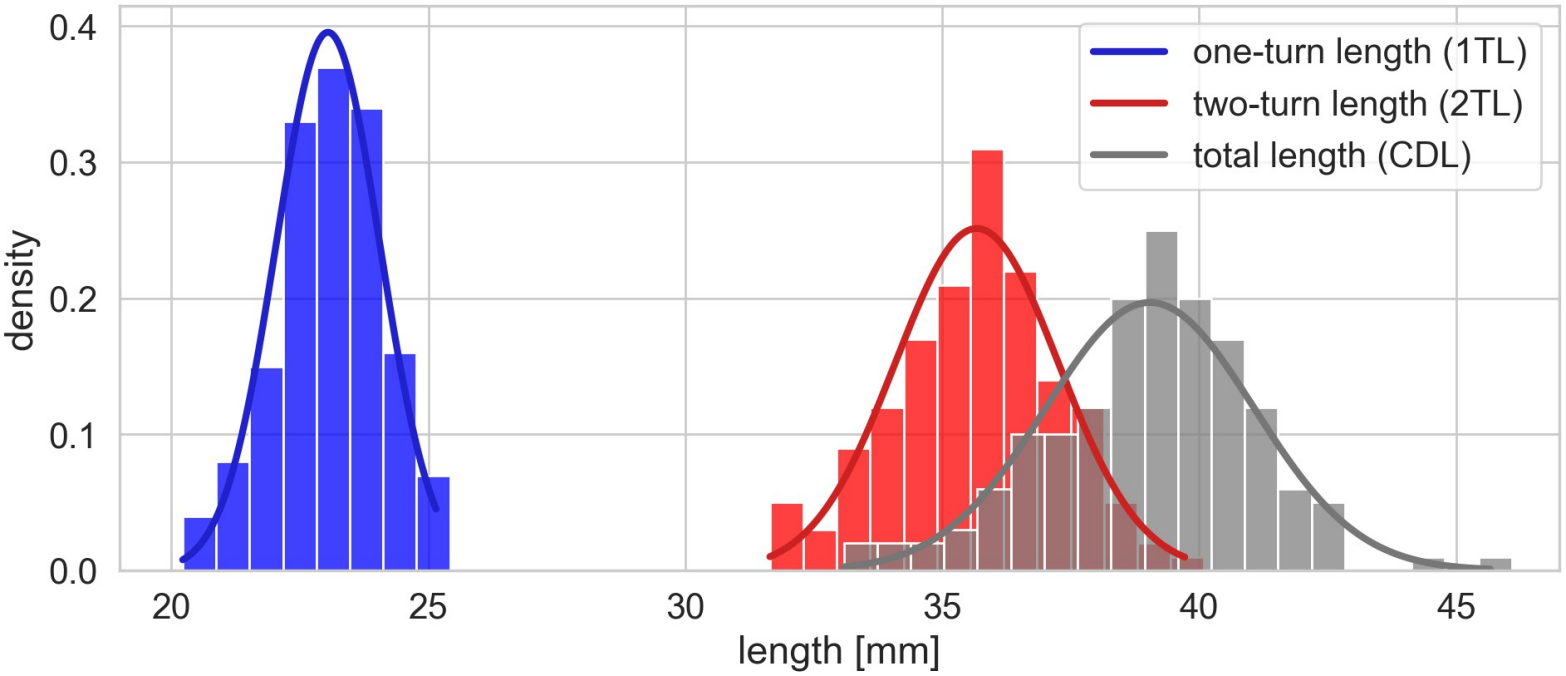
Histograms of cochlear length measurements with fitted normal distributions: One-turn length (1TL, blue), two-turn length (2-TL, red) and total cochlear duct length (CDL, gray).

In the cohort described above, a mean cochlear length of 39.04mm (+/- 2.03mm standard deviation) was measured. The mean one-turn length (1TL, length of the lateral wall in the basal turn, i.e. 360° from the reference point in the round window) and the mean two-turn length (2TL, length of the lateral wall in the first two turns from the round window) were 23.05mm (+/- 1.01mm) and 35.67mm (+/- 1.59mm) respectively (see Fig 2). The mean first turn’s contribution to the total length of the cochlea was 59.1% (+/- 2%-points), while the mean second turn’s contribution was on 32.3% (+/- 1.1%-points).

**Fig 3:**
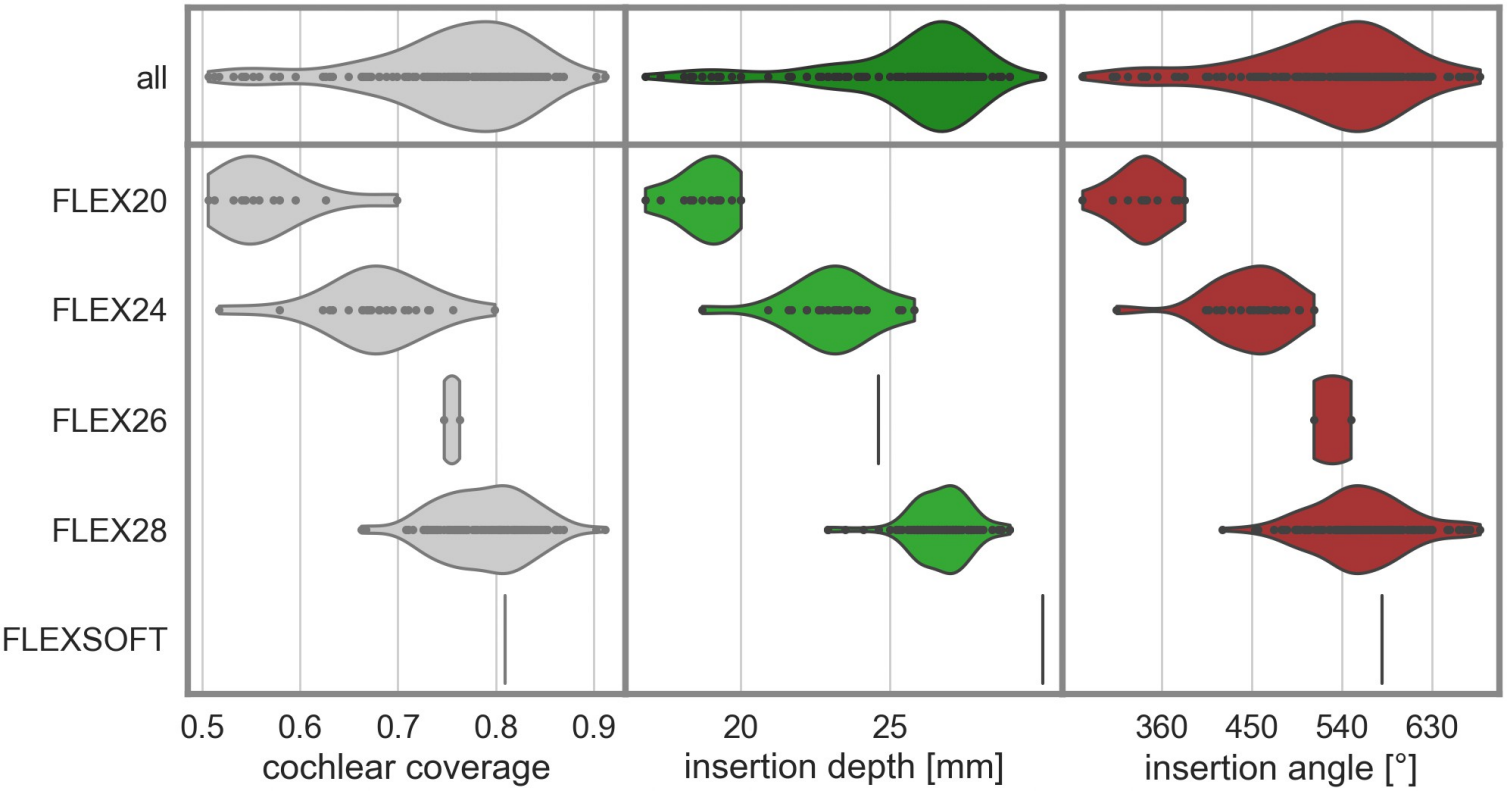
Distributions of cochlear coverage (left), insertion depth (center), and insertion angle (right) across the entire cohort (top row) and across sub-populations with different electrode arrays (bottom panels).

The mean IA was 524° (+/- 82°), the mean insertion depth was 25.46mm (+/- 2.68mm), and the mean CC was 75.2% (+/- 8.5%-points). There was a strong correlation between CC and IA (Pearson r = 0.95). The overall distribution of these metrics and their distribution with respect to the different electrode arrays present in the studied cohort are shown in Fig 3. The mean values and ranges for the different electrode arrays are given in Table 1.

### Speech recognition test scores

Pre-operative FMWT_pre_ scores were acquired on average 45 days before the surgery. Post-operative FMWT_post_ and HSM scores were acquired as part of the 12 months post-operative rehabilitation appointment, which was scheduled on average 375 days after the initial fitting. Mean values and ranges for the different electrode arrays are given in Table 1.

FMWT_post_ scores were available for all 154 ears. A significant correlation was found between FMWT_post_ scores and both CC (Pearson r = 0.233, p = 0.004) as well as IA (r = 0.221, p = 0.006). In a linear regression model, FMWT_post_ scores increased by 6%-points per 10%-points CC, and in a separate linear regression model FMWT_post_ scores increased by 5.5%-points per 90° IA. Subsequently, a multivariate regression analysis including the age at implantation, the duration of hearing impairment before implantation, and the post-operative low-frequency PTA as potential confounding factors (N = 147 due to seven missing low-frequency PTA values) showed that CC, age, and duration of hearing impairment were independently significant factors in the model (p = 0.004, p<0.001, and p = 0.046 respectively), while post-operative low-frequency PTA was not a significant factor (p = 0.702). The overall model explained 17.7% of the variance in FMWT_post_ scores. In a second model including IA instead of CC, IA, age, and duration of hearing impairment were significant factors (p = 0.008, p<0.001, and p = 0.045), while post-operative low-frequency PTA was again not a significant factor. Here, the overall model explained 17.1% of the variance in FMWT_post_ scores. Of these 147 ears, pre-operative FMWT_pre_ scores were missing at random for 26 ears, and the multivariate regression analysis was repeated for the sub-group of the remaining 121 ears, additionally including FMWT_pre_ scores as an independent factor. In this linear regression model, CC, age, and FMWT_pre_ scores were independently significant factors (p = 0.001, p<0.001, and p<0.001), while duration of hearing impairment (p = 0.073) and post-operative low-frequency PTA (p = 0.316) were not significant factors. The overall model now explained 34.4% of the variance in FMWT_post_ scores. When replacing CC with IA in the model, IA, age, and FMWT_pre_ scores were significant factors (p = 0.002, p<0.001, and p<0.001) and the overall model explained 33.2% of the variance in FMWT_post_ scores. Including interaction terms between CC and either of the other independent factors in any of these models did not reveal any significant interactions.

HSM sentence test scores in noise were only available for 143 of 154 ears due to time constraints in the clinical routine. A significant correlation could not be found between HSM scores and neither CC nor IA (p = 0.341 and p = 0.279, respectively). Consequently, a multivariate regression analysis was not carried out.

**Fig 4:**
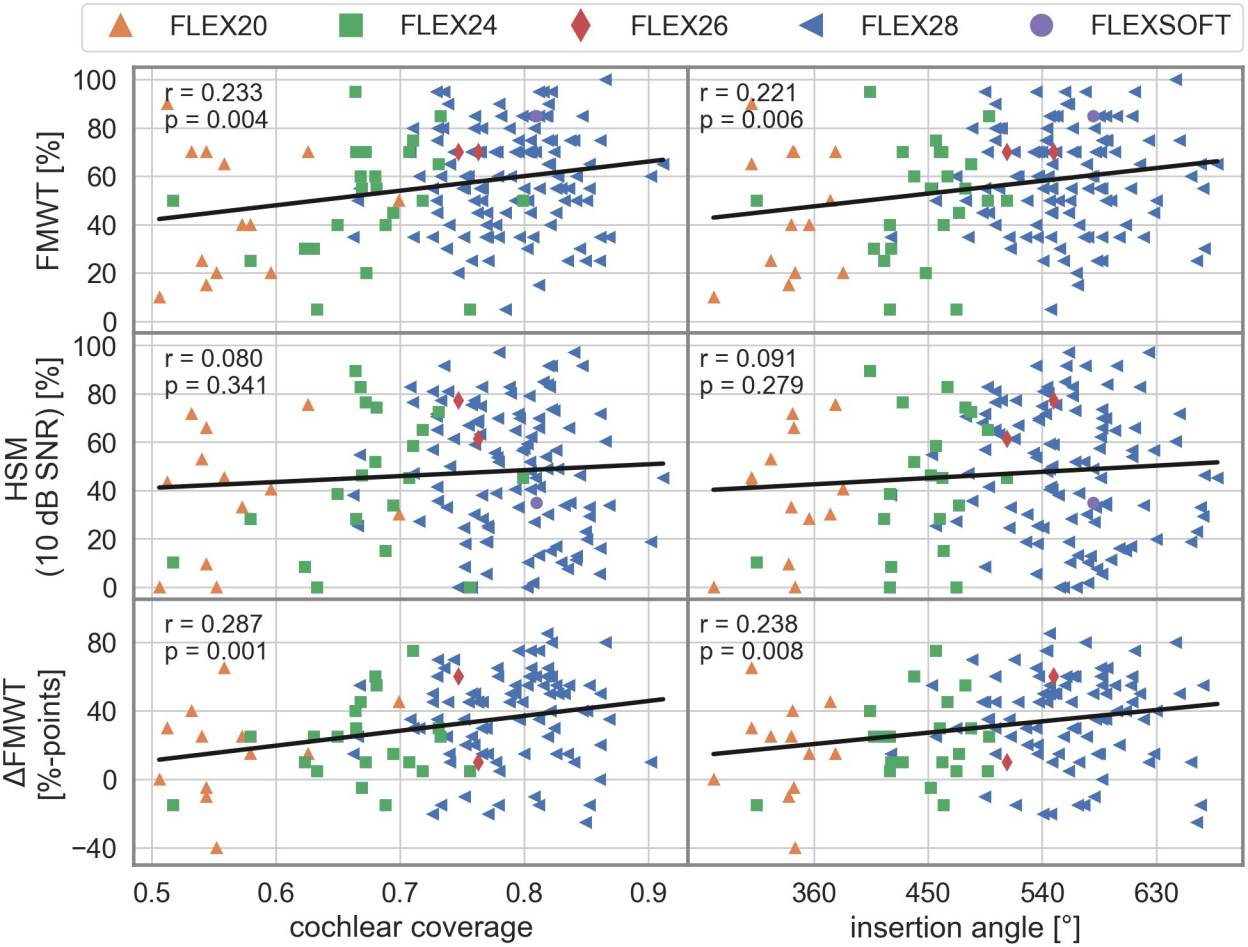
Scatter plots of FMWT scores (top row), HSM sentence test scores in noise (center row) and ΔFMWT (bottom row) over cochlear coverage (left column) and insertion angle (right column). Solid lines, r-, and p-values show the results of linear regression modeling. Different symbols correspond to different electrode arrays.

FMWT_pre_ were available for 125 ears of the cohort, and in this sub-group both CC (r = 0.287, p = 0.001) as well as IA (r = 0.238, p = 0.008) were significantly correlated with the difference between post- and pre-operative FMWT scores (ΔFMWT). In a linear regression model, ΔFMWT increased by 8.7%-points per 10%-points CC, and in a separate linear regression model ΔFMWT increased by 6.6%-points per 90° IA. The following multivariate regression analysis including age at implantation, duration of hearing impairment before implantation, post-operative low-frequency PTA, and FMWT_pre_ scores (N = 121) showed that CC, age, and FMWT_pre_ scores were all independently significant factors (p = 0.001, p<0.001, and p<0.001), while duration of hearing impairment and post-operative low-frequency PTA were not statistically significant factors (p = 0.073 and p = 0.316). The overall model explained 55.4% of the variance in ΔFMWT. In a second model with IA replacing CC, IA, age, and FMWT_pre_ scores were significant factors (p = 0.002, p<0.001, and p<0.001), while duration of hearing impairment and post-operative low-frequency PTA were again not significant factors (p = 0.077 and p = 0.323). Here, the overall model explained 54.5% of the variance in ΔFMWT. Fig 4 shows the corresponding scatter plots and linear regression modeling results.

It has been reported previously that deeper insertions above a certain threshold provide no further benefit with respect to speech recognition with a CI [25]. Therefore, in order to further investigate if there were any other relationships between CC and test scores that would not be captured by a linear regression model, we divided the cohort into five groups based on the quintiles of CC, resulting in limits between groups at CC of 70%, 75%, 79%, and 82%. A Kruskal-Wallis test showed a significant effect of CC quintile on FMWT_post_ scores (H(4) = 14.39, p = 0.006), but no significant effect of CC quintile on HSM scores (H(4) = 4.8, p = 0.31). Pairwise comparisons using Dunn’s test with Holm-Bonferroni correction indicated that mean FMWT_post_ scores between the first quintile of CC (CC < 70%) and the fourth quintile of CC (79% < CC < 82%) were statistically different (p = 0.003, means scores: 46% vs 66%). No other differences were statistically significant.

**Fig 5:**
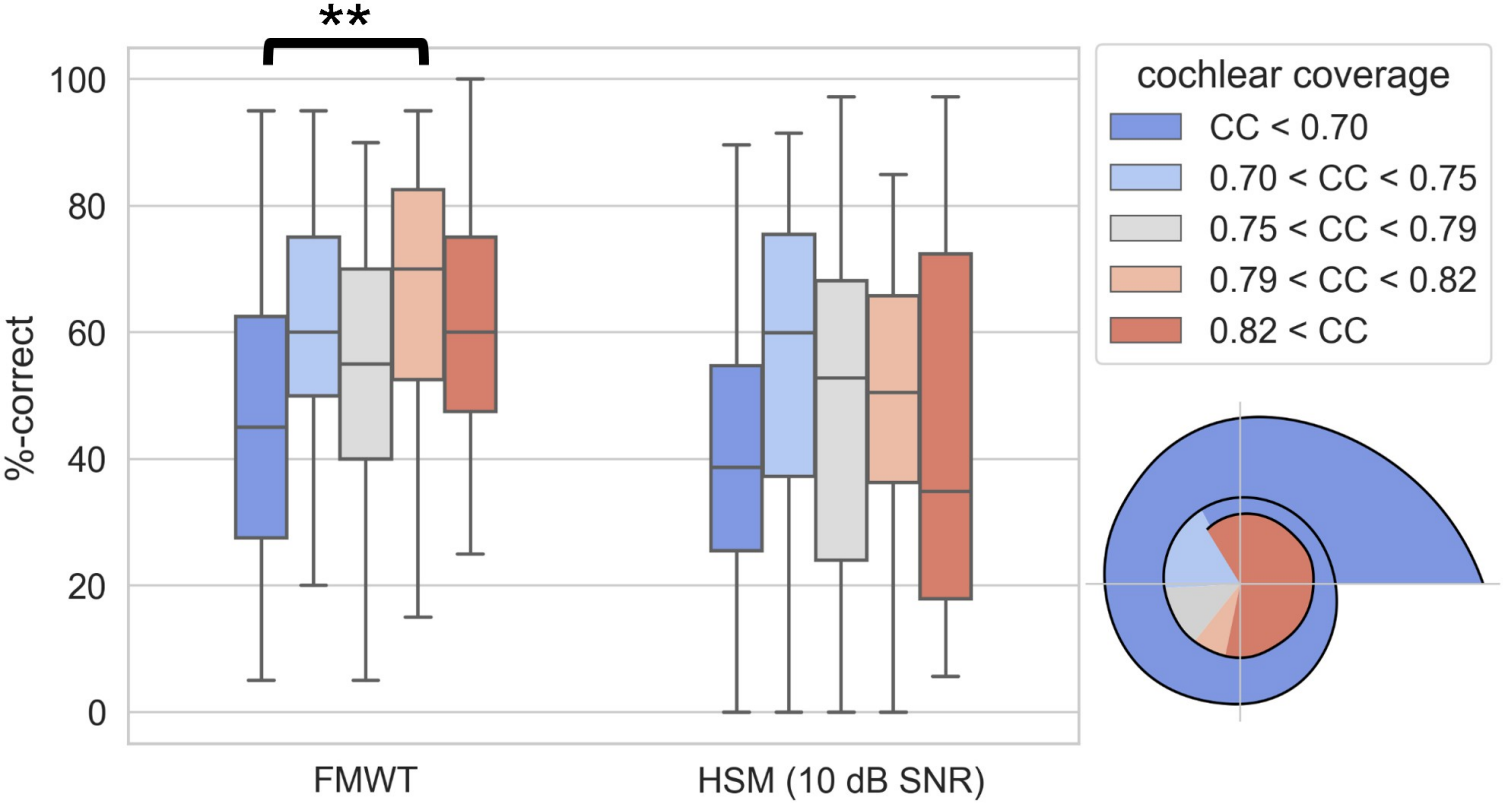
Box plot of FMWT scores (left) and HSM sentence test scores in noise (right) for groups of subjects based on cochlear coverage quintiles. Colored areas in the diagram in the bottom right corner correspond to the ranges of cochlear coverage of the five quintiles in an average cochlea. The whiskers indicate the minimum and maximum value in each group. (*: p<0.05; **: p<0.01; ***: p<0.001).

**Fig 6:**
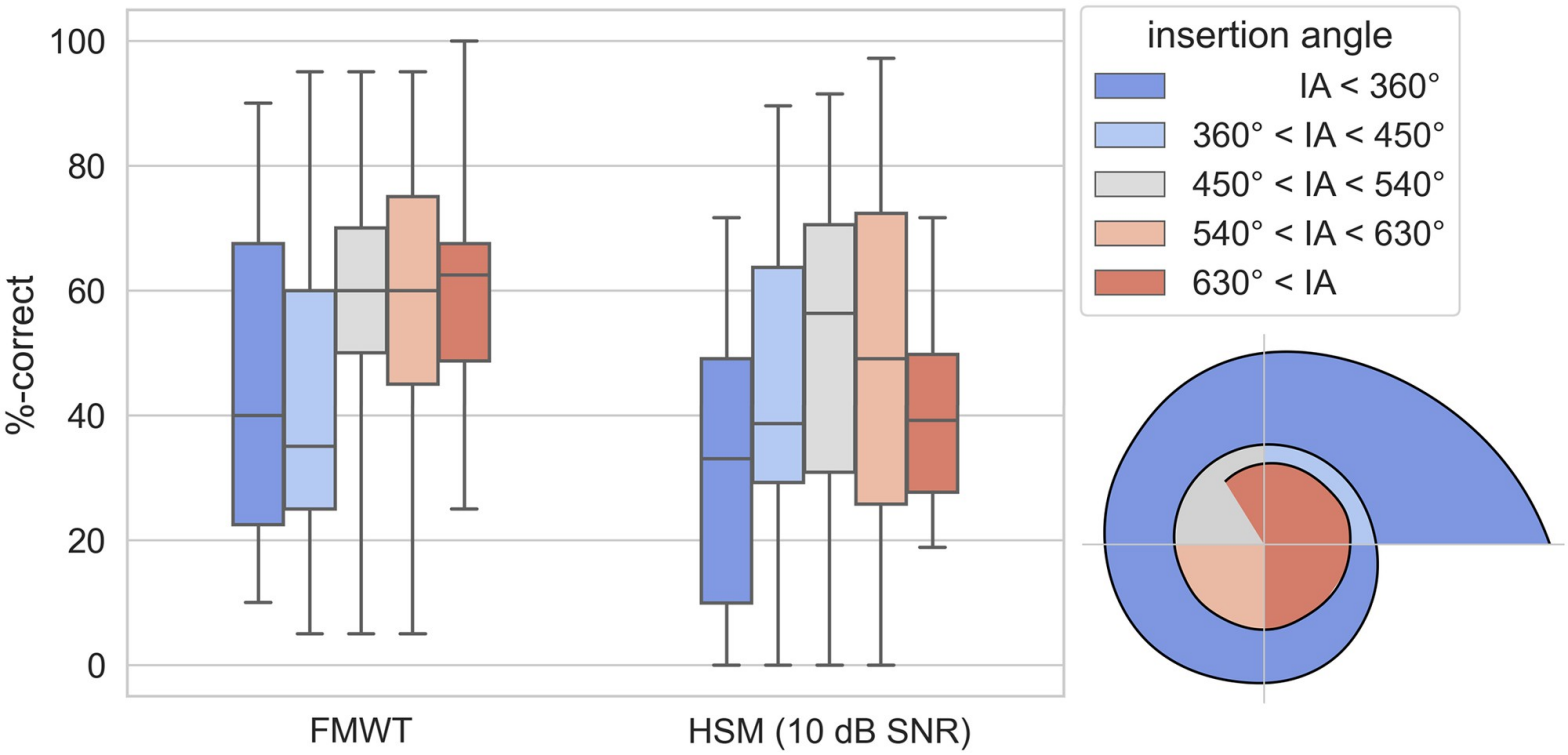
Box plot of FMWT scores (left) and HSM sentence test scores in noise (right) for groups of subjects based on insertion angle quadrants. Colored areas in the diagram in the bottom right corner correspond to the ranges of insertion angle of the five groups. The whiskers indicate the minimum and maximum value in each group. (*: p<0.05; **: p<0.01; ***: p<0.001).

For a group-wise analysis of the effect of IA, we chose a more intuitive approach and divided the cohort into five groups based on landmarks in regular intervals of 90°, starting at 360° from the reference point in the center of the round window. A Kruskal-Wallis test for the effect of IA group on FMWT_post_ scores showed statistical significance (H(4) = 9.58, p = 0.048), however no significant differences between groups were found in pairwise comparisons using Dunn’s test with Holm-Bonferroni correction. A Kruskal-Wallis test for the effect of IA group on HSM scores did not reach statistical significance (H(4) = 5.078, p = 0.279). Speech recognition scores in groups based on CC are shown in Fig 5, and speech recognition scores in groups based on IA are shown in Fig 6.

**Fig 7:**
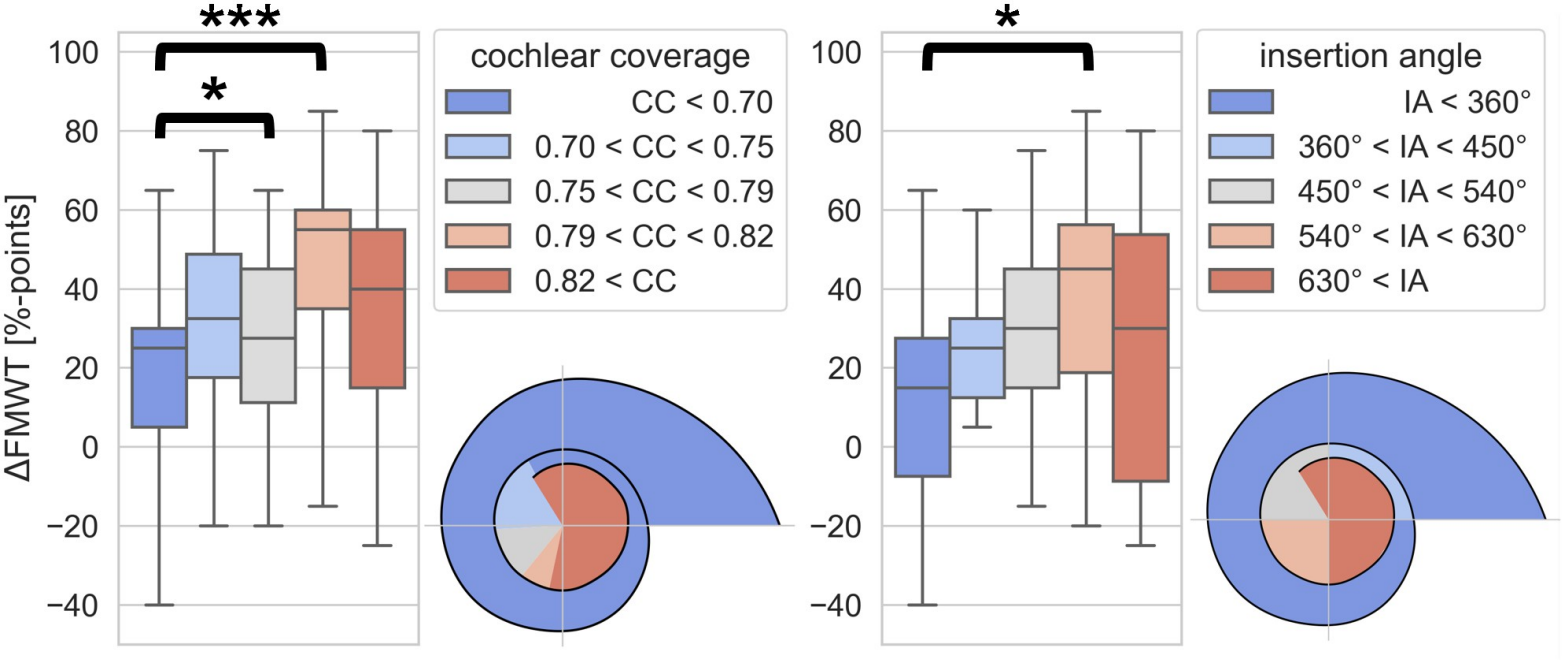
Box plot of ΔFMWT for groups of subjects based on cochlear coverage quintiles (left side) and for groups of subjects based on insertion angle quadrants (right side). Colored areas in the diagrams correspond to the ranges of cochlear coverage of the five quintiles, and the ranges of insertion angle of the five groups, respectively. The whiskers indicate the minimum and maximum value in each group. (*: p<0.05; **: p<0.01; ***: p<0.001).

Using the same method to group subjects as above, we also analyzed the effect of CC and IA on ΔFMWT. A Kruskal-Wallis test showed a significant effect of both CC group (H(4) = 17.58, p = 0.001) as well as IA group (H = 10.37, p = 0.035) on ΔFMWT. Pairwise comparisons between CC groups using Dunn’s test with Holm-Bonferroni correction indicated that mean ΔFMWT was significantly different between the first and the fourth quintile of CC (19.5 vs 47.5%-points, p<0.001), as well as between the third and the fourth quintile of CC (26.5 vs 47.5%-points, p = 0.036). Pairwise comparisons between IA groups indicated that mean ΔFMWT was significantly different between group 1 and group 4 (11.8 vs 38.5%-points, p = 0.049). ΔFMWT values in groups based on CC and in groups based on IA are shown in Fig 7. Cohort and group sizes for the linear regression modeling and both methods of grouping subjects are given in Table 2.

**Table 2:** Cohort sizes for the different statistical analyses.

		Group limits	N (FMWT)	N (HSM)	N (ΔFMWT)
Linear regression model		-	154	143	125
**CC-based division**	Group 1	**CC** < 0.70	31	29	29
	Group 2	0.70 < **CC** < 0.75	31	26	26
	Group 3	0.75 < **CC** < 0.79	30	27	22
	Group 4	0.79 < **CC** < 0.82	31	30	23
	Group 5	0.82 < **CC**	31	31	25
**IA-based division**	Group 1	**IA** ≤ 360°	11	11	11
	Group 2	360° < **IA** ≤ 450°	13	11	11
	Group 3	450° < **IA** ≤ 540°	49	46	41
	Group 4	540° < **IA** ≤ 630°	73	67	56
	Group 5	630° < **IA**	8	8	6

Cohort sizes for linear regression modeling (top row) and group sizes for the group-wise analysis after the division based on cochlear coverage (CC) quintiles and after the division based on insertion angle (IA) quadrants.

We also analyzed if any of the potential confounding factors were different between CC groups and/or IA groups, respectively. Separate Kruskal-Wallis tests for age at implantation, duration of hearing impairment, and pre-operative FMWT scores revealed that neither CC group (H = 7.127, p = 0.129; H = 2.526, p = 0.64; H = 1.505, p = 0.826) nor IA group (H = 2.089, p = 0.719; H = 2.927, p = 0.57; H = 2.806, p = 0.591) had an effect on these factors, showing that they were not significantly different between groups. A Kruskal-Wallis test for post-operative low-frequency PTA, however, showed a significant effect on CC (H = 21.001, p<0.001) as well as IA (H = 19.438, p = 0.001). Pairwise comparisons between CC groups using Dunn’s test with Holm-Bonferroni correction showed that post-operative low-frequency PTA was signficantly lower in the first quintile of CC than in the fourth quintile (p = 0.012) and fifth quintile (p<0.001) of CC. Pairwise comparisons between IA groups showed that post-operative low-frequency PTA was signficantly lower in the first IA group than in group 5 (p = 0.034), and also lower In IA group 2 than in groups 4 (p = 0.01) and 5 (p = 0.01).

Since there were a total of 22 ears with disabled electrode contacts (17 with complete insertions and 5 with incomplete insertions) we repeated the analysis in two sub-groups of the cohort: In sub-group 1 we excluded the five ears with incomplete insertions, and in sub-group 2 we excluded all 22 ears with disabled electrode contacts. We repeated the linear regression analysis between CC/IA and the three outcomes measures FMWT scores, HSM scores, and ΔFMWT, as well as the group-based comparison of these outcomes measured between groups of different CC/IA. The limits for the division into groups were kept unchanged from the ones used for the full cohort (see Table 2). The results of the analysis in the two sub-groups are shown in Table 3. The linear regression analyses largely led to the same results as for the full cohort, showing positive correlations between CC/IA and FMWT_post_ scores and ΔFMWT, respectively, while showing no significant correlation between CC/IA and HSM scores. The group-wise comparisons in sub-group 1 also led to the same results as for the full cohort with the exception of ΔFMWT no longer being significantly different between any IA groups. In sub-group 2, in addition to the significant differences already observed in the full cohort, FMWT_post_ scores were significantly higher in the second CC quintile than in the first CC quintile, and also significantly higher in IA groups 3 and 4 than in IA group 2. However, similar to sub-group 1, there were no longer any significant differences in ΔFMWT between IA groups.

**Table 3:** Results of the analysis in sub-groups with extracochlear and deactivated electrode contacts.

	**linear regression**	**linear regression**	**linear regression**
	**CC → FMWT**	**CC → HSM**	**CC → ΔFMWT**
**Sub-group 1**	r = 0.192 (N = 149)	r = 0.015 (N = 138)	r = 0.243 (N = 120)
	p = 0.031*	p = 0.858	p = 0.008**
**Sub-group 2**	r = 0.264 (N = 132)	r = 0.074 (N = 123)	r = 0.262 (N = 105)
	p = 0.002**	p = 0.414	p = 0.007**
	**linear regression**	**linear regression**	**linear regression**
	**IA → FMWT**	**IA → HSM**	**IA → ΔFMWT**
**Sub-group 1**	r = 0.177 (N = 149)	r = 0.029 (N = 138)	r = 0.191 (N = 120)
	p = 0.031*	p = 0.732	p = 0.037*
**Sub-group 2**	r = 0.255 (N = 132)	r = 0.093 (N = 123)	r = 0.205 (N = 105)
	p = 0.003**	p = 0.304	p = 0.036*
	**Group-wise comparison in CC groups**		
	**FMWT**	**HSM**	**ΔFMWT**
**Sub-group 1**	Kruskal-Wallis:	Kruskal-Wallis:	Kruskal-Wallis:
	H = 11.6, p = 0.021*	H = 3.48, p = 0.481	H = 15.499, p = 0.004**
	group 1 vs group 4:		group 1 vs group 4:
	p = 0.014*		p = 0.003**
			group 3 vs group 4:
			p = 0.044*
**Sub-group 2**	Kruskal-Wallis:	Kruskal-Wallis:	Kruskal-Wallis:
	H = 15.829, p = 0.003**	H = 6.564, p = 0.161	H = 14.143, p = 0.007**
	group 1 vs group 2:		group 1 vs group 4:
	p = 0.018*		p < 0.001***
	group 1 vs group 4:		
	p = 0.001**		
	**Group-wise comparison in IA groups**		
	**FMWT**	**HSM**	**ΔFMWT**
**Sub-group 1**	Kruskal-Wallis:	Kruskal-Wallis:	Kruskal-Wallis:
	H = 6.862, p = 0.143	H = 2.592, p = 0.628	H = 7.141, p = 0.129
**Sub-group 2**	Kruskal-Wallis:	Kruskal-Wallis:	Kruskal-Wallis:
	H = 13.847, p = 0.008**	H = 6.109, p = 0.191	H = 7.476, p = 0.113
	group 2 vs group 3:		
	p = 0.022*		
	group 2 vs group 4:		
	p = 0.022*		

Results of the linear regression analysis and the group-wise analysis for two different sub-groups of the cohort: sub-group 1 consists of ears with complete insertions only (N = 149), and sub-group 2 consists of ears with all 12 electrode contacts active (N = 132). The top two sections of the table show the results of the linear regression analysis. The bottom two sections of the table show the results of the group-wise analysis. Differences between groups are only shown in the table if they were significant in pairwise comparisons using Dunn’s test with Holm-Bonferroni correction (*: p < 0.05, **: p<0.01, ***: p < 0.001).

## Discussion

### Cochlear geometry and implant insertion depth

Previously reported measurements of the cochlear length vary substantially depending on the available imaging data and the measurement method that is applied. Skinner et al. [17] for example reported a mean cochlear length of 34.62 mm including the hook region, while Erixon et al. [31] reported a mean cochlear length of 42 mm starting from the center of the round window. The imaging data and measurement method used by Timm et al. [26] were similar to those used in the present study and they reported a mean cochlear length of 37.9 mm (+/- 2.4 mm). Eser et al. [32] also used similar data and methods and reported a mean cochlear length of 42.09 mm (+/- 1.17 mm) in a group of normal hearing subjects. As was pointed out in these studies, it is sometimes difficult to determine the exact location of the lateral wall due to the quality of clinical CBCT scans, resulting in the lateral wall showing as a gradient area rather than a sharp edge. Therefore, small differences between mean cochlear lengths reported by different observers should not be surprising and the values measured in this study are well within the range of previous results. Erixon et al. [31] also measured the proportional length of each cochlear turn, with the first turn (starting from the round window) representing on average 53% of the total length, the second turn representing on average 30% of the total length, and the apical turn representing on average 17% of the total length. In the current studies, the mean values were 59%, 32.3%, and 8.6% of the total length for the first, second and apical turn, respectively. The discrepancies between these values are most likely artifacts of the measurement method, as larger values for the first turn may arise if the trace of the lateral wall is drawn slightly more laterally.

The measured IA values are in line with those reported by previous studies [25, 33, 34]. Very few data have been published on proportional cochlear coverage measurements. Skinner et al. [17] reported a mean cochlear coverage of 55%, however all patients in their cohort were implanted with a lateral wall electrode array with a length of 25mm (Nucleus 22, Cochlear, Australia). Cooperman et al. [24] estimated CC in a cohort with FLEX electrode arrays based on CDL measurements and the electrode array length provided by the manufacturer. They reported a mean CC of 76%, which is remarkably similar to the results in this study. However, since they did not verify the actual electrode array position in post-operative imaging data and the distribution of electrode arrays (FLEX24: 54.10%, FLEX28: 32.79%, FLEXSOFT/Standard: 13.11%) was substantially different to that of the cohort analyzed in the present study, it is difficult to compare CC values between the two studies.

### Speech recognition

We decided to investigate the effect of both IA as well as CC on post-operative speech recognition. The IA is easier to determine and already captures some of the individual differences in cochlea size (thus being preferable to linear insertion depth or electrode array length alone), and has therefore been used in the majority of previous studies on the topic. Unlike CC, however, it does not take into account individual differences in CDL (including apical differences). Under the assumption that CDL is a more meaningful indicator of the individual extent of the organ of Corti [35, 36], it could be expected that CC is a more appropriate value to examine the effect of electrode array position on CI outcomes. Nevertheless, in the results of this study, the measured effects of CC and IA on post-operative speech recognition scores were largely the same. Since any difference between the effect of CC and IA would be expected to be most pronounced for very deep insertions where the individual extent of the organ of Corti could make a difference in speech recognition, it is possible that there simply were not enough samples of insertions that deep, to create a notable difference.

We found that post-operative FMWT scores at 12 months after activation were positively correlated with both CC and IA. Even after combining CC (or IA) with other important factors in a multivariate regression model, it was confirmed as an independently significant factor for FMWT scores. Compared to previous results, we found a similar correlation coefficient and a similar slope in the regression analysis as O’Connell et al. [20]. However, their cohort was much more diverse than the one in this study with respect to electrode array types, surgical approach, and scalar dislocations. Other studies analyzed more similar, albeit smaller, cohorts and found slightly stronger correlations between word recognition scores and IA than the one observed in our results [19, 21, 25]. It is not quite clear why the correlation between IA and word recognition scores observed in the present study was weaker than in those previous studies. At least in the study published by Canfarotta et al. [25] a smaller range and lower average values of duration of hearing loss were reported, which could at least partly account for the different correlations. Generally, the cohort analyzed in the present study covered a wide range of age at implantation and duration of hearing loss, which may have contributed to a wider spread of speech recognition scores. Additionally, the majority of ears analyzed in the present study had been implanted with FLEX28 electrode arrays, and in consequence IA values were more frequent in the range typical for that electrode array. Potentially, a stronger correlation between CC/IA and FMWT scores might have been observed if both shorter and longer electrode arrays had been more frequent in the cohort.

When dividing the cohort into five equally large groups based on CC, FMWT scores were highest in the group of patients with CC between 79%-82%. Only the difference to the group of patients with CC below 70% was statistically significant. Nevertheless, median FMWT scores of patients with CC between 70%-79% and patients with CC above 82% were at least 10%-points lower than group with the highest performance. A significant effect of IA on FMWT scores was observed when comparing groups based on IA. Post-hoc pairwise comparisons, however, did not reveal any significant differences between groups, even though median FMWT scores were at least 20%-points lower for patients with IA below 450° than for patients with IA above 450°. It can be speculated that the lack of statistical significance in spite of strong differences in median performance is at least partly due to highly unequal group sizes that resulted from creating groups based on IA quadrants.

Interestingly, not only the absolute post-operative FMWT scores, but also the difference between post- and pre-operative scores ΔFMWT was positively correlated with measures of insertion depth. This implies that patients with deeper insertions did not only reach a higher level of speech recognition after surgery, but that they also experienced higher gains due to surgery. It should, however, be noted that pre-operative FMWT scores were measured in the unaided condition using headphones, and post-operative FMWT scores were measured in the free field condition. Thus, ΔFMWT should not be interpreted as a literal improvement (in that ΔFMWT<0 does not mean that post-operative word recognition performance was worse than pre-operative word recognition performance). However, it serves as a good indicator of the benefit patients received with respect to their pre-operative state.

A potential reason for the positive correlation between deeper insertions and CI performance lies in the improved utilization of the cochlear structures. It has been shown that spiral ganglion cell count affects post-operative word recognition scores [37, 38]. Therefore, it could be assumed that stimulation with a CI is more successful if a larger amount of spiral ganglion cells is targeted. Furthermore, it has been speculated that better speech recognition abilities after deeper insertions are linked to the reduced frequency mismatch between the natural tonotopy of the inner ear and the default frequency-to-electrode allocation of the CI [12, 19, 34]. Even though it has been shown that CI patients are able to compensate for large frequency mismatches [39], the individual extent of adaptation varies and may remain incomplete (e.g. [40, 41]). Consequently, shallower insertions that produce a large frequency mismatch and thus impede the adaptation process might impair CI outcomes. Nevertheless, the major part of the learning process with a CI happens within the first 12 months after implantation, which is when the speech recognition data for this study was measured, and insertion depth was shown previously to not have a significant effect on the duration of the learning process [42]. Even though we restricted implants in this this study to those of only one manufacturer to avoid the influence of differences in electrode array design, we still included electrode arrays of different lengths and different electrode contact spacing. Since increased contact spacing has been associated with reduced channel interaction [34], the larger contact spacing of longer electrode arrays may also have contributed to the positive correlation between higher CC/larger IA and word recognition scores.

While both CC and IA were found to be significantly correlated with FMWT scores, no significant correlation was found between CC or IA and HSM scores. In the group-wise comparison of groups based on CC quintiles, CI performance was lower for subjects in the fifth CC quintile compared to the fourth CC quintile, with median HSM scores in the fifth quintile being even lower than those in the first quintile. When dividing the cohort based on IA quadrants FMWT scores for IA below 450° was lower than for larger IA. A similar tendency showed for HSM scores, but a drop at very high IA above 630° was observed additionally. Due to the lack of statistical significance, these findings should be treated carefully. However, a lack of additional benefit or even a negative effect of very deep insertions on CI performance has been reported previously [24, 25, 43]. It has been speculated before, that increased frequency-to-place mismatch may contribute to reduced performance in word and sentence recognition tests (eg. [44, 45]). However, for the electrode array types analyzed in this study, deeper insertions generally reduce the frequency-to-place mismatch except for extreme cases with exceptionally deep insertions [34]). Another possible cause of reduced CI performance after very deep insertions may be the decreased coverage of the basal part of the cochlea. Previously, evidence was found that deeper insertion of the most basal electrode contact might negatively affect speech perception outcomes of CI recipients [10, 23]. Therefore, we also determined the linear distance between the most basal electrode contact and the reference point in the round window. Since one or more intracochlear electrode contacts had been disabled in the fitting for 17 subjects of the cohort, we always measured the distance between the round window and the most basal electrode contact that was also active in the fitting. The mean distance was 2.58mm (+/- 0.94mm standard deviation), and no significant correlation could be observed between the distance and any of the three outcome measures FMWT, HSM, and ΔFMWT.

A further potential reason for decreased CI performance after very deep insertions is given by a higher risk of trauma. The scala tympani becomes increasingly narrow towards the helicotrema, with the cross-sectional area typically falling below 1 mm^2^ for angles greater than 540° from the round window [46]. Therefore, very deep insertions pose a higher risk of introducing trauma that could negatively affect the electrode-to-nerve interface and hence also impair the patient’s speech recognition ability. It has been shown previously that insertional trauma caused a higher amount of new bone in the cochlea, which in turn was negatively correlated with post-operative CNC word recognition scores [38].

Finally, CI performance might be lower after very deep insertions due to a larger amount of crosstalk, where the same neuronal structures are recruited by two or more electrode contacts. Firstly, the spatial distance between two electrode contacts is smaller in the apical region of the cochlea because the radius of the apical turn is smaller than the radius of the basal turn. Secondly, the peripheral dendrites do not extend radially from the basilar membrane to the spiral ganglion cells above a certain point towards the apex [47]. Consequently, spiral ganglion cells are more densely packed in the apical region of the cochlea, increasing the risk of crosstalk between electrode contacts. Patients with very deep insertions have even been shown to benefit from deactivating the two or three most apical electrode contacts [43].

A total of 22 implants in the study cohort had at least one electrode contact disabled, and five of these implants were not inserted completely, leaving one or two extracochlear electrode contacts. Except for one implant where contact 6 was disabled, only the most basal one or two electrode contacts were disabled. Even though results of previous studies suggest that speech understanding would not be affected by the de-activation by one or two basal electrode contacts [13], we repeated the linear regression analysis and the group-wise comparisons for two sub-groups of the cohort. In the first sub-group only the 5 ears with incomplete insertions were excluded, while in the second subgroup all 22 ears with disabled electrode contacts were excluded from the cohort. Overall, there were no major differences in the analysis results of those sub-groups compared to the results of the full cohort, which suggests that a slightly reduced number of active electrode contacts was not a contributor to the observed correlations between CC/IA and word recognition scores.

Based on available findings at the time, both Mistrík and Jolly [48] as well as Timm et al. [26] recommended a CC of 80% to ensure optimal outcomes. Based on the results of the group-wise comparison of post-operative FMWT scores in this study, CC above 70% or an IA above 450° should be preferred. Based on the comparison of ΔFMWT between groups, preferred CC should be above 79%, and the preferred IA should be above 540°. We found that the median scores in all three outcome measures decreased again for insertions deeper than 82% CC, and that median HSM scores and the median ΔFMWT decreased again for insertions deeper than 630° IA, even though these differences were not statistically different. When using these findings as indication limits, the resulting range of 79%-82% CC is remarkably similar to the previous recommendations mentioned above, and the resulting range of 540°-630° IA is in line with the results of Canfarotta et al. [25], who observed no further benefit of insertions deeper than 600°. For a cochlear coverage of 80%, Timm et al. [26] derived that the FLEX24 would be indicated for a CDL of 32.9mm, the FLEX28 would be indicated for a CDL of 38.2mm, and the FLEXSOFT would be indicated for a CDL of 42.7mm. The majority of patients (given electric-only stimulation) would consequently be best treated with the FLEX26, FLEX28 or FLEXSOFT electrode arrays depending on their CDL, while only patients with very small cochleae would be best treated with a FLEX24 electrode array.

### Limitations of the study

Several studies have shown that there is a sizeable correlation between daily processor wear time and both word and sentence recognition (e.g. [49, 50]). Unfortunately, in the present study’s cohort, data about the subjects’ processor wear time was not available in a manner consistent enough for a meaningful analysis. Missing data was either due to older processor models that did not track processor wear time or due to inconsistencies in the database. Therefore, an influence of daily processor wear time on the results of this study cannot be excluded.

Furthermore, it has been shown in the past that scalar translocations of the electrode array (i.e. the electrode array puncturing the basilar membrane and deviating into the scalar vestibuli) have a negative effect on post-operative speech recognition (e.g. [9, 20]). With the analysis method applied in the present study, it is not possible to reliably detect scalar translocations of the electrode array. However, ears included in this study were all implanted with lateral wall electrode arrays using the round window approach. In a review of studies analyzing surgical procedures, Jwair et al. [51] found scalar translocations in only 2% of the implantations of lateral wall electrode arrays using the round window approach. Consequently, while it is unlikely that they were frequent enough to substantially affect the results, it cannot be ruled out that some implants with scalar translocation were present in the studied cohort.

Finally, the lateral wall and electrode array tracings in the CBCT images were done by only one observer, and the fidelity of the observer was not measured. However, the resulting values of cochlear size, cochlear coverage and insertion angle were in similar ranges as results of previously reported studies.

## Conclusions

In this study, CC and IA were measured in 154 ears treated with a CI with lateral wall electrode arrays of different length. In contrast to previous studies with cohorts of similar size, the cohort analyzed in this study was uniform with respect to electrode array type and surgical approach. Subsequently, the relationship between CC/IA and post-operative speech recognition scores 12 months after the initial fitting of the device was examined. The results showed a positive linear correlation between CC/IA and post-operative FMWT scores independently of other factors commonly associated with CI outcomes. Additionally, a positive linear correlation was observed between CC/IA and ΔFMWT, indicating that patients with deeper insertions perform better in speech recognition tasks and also had a larger benefit of CI with respect to their pre-operative state. The results of a group-wise comparison of FWMT scores, HSM scores, and ΔFMWT showed that median scores were highest in a range between 79%-82% for CC and between 540°-630° for IA, respectively. A customized choice of electrode array during pre-operative planning on a patient-individual basis therefore has the potential to maximize the probability of optimized CI outcomes.

## Supporting information

S1 File Regression analysis results. (DOCX)

S2 File Data. (XLSX)
