# Supplementary material for: Cochlear coverage with lateral wall cochlear implant electrode arrays affects post-operative speech recognition
Source: PLoS One. 2023 Jul 12;18(7):e0287450. doi: 10.1371/journal.pone.0287450 (PMC10337941; doi:10.1371/journal.pone.0287450)
Supplement: S1 File — (DOCX) [file pone.0287450.s001.docx]

Independent variable: CC; dependent variable: FMWT_post_

OLS Regression Results

==============================================================================

Dep. Variable: fmt_M12 R-squared: 0.054

Model: OLS Adj. R-squared: 0.048

Method: Least Squares F-statistic: 8.746

Date: Tue, 26 Jul 2022 Prob (F-statistic): 0.00360

Time: 15:30:45 Log-Likelihood: -688.88

No. Observations: 154 AIC: 1382.

Df Residuals: 152 BIC: 1388.

Df Model: 1

Covariance Type: nonrobust

==============================================================================

coef std err t P>|t| [0.025 0.975]

------------------------------------------------------------------------------

const 11.7750 15.426 0.763 0.446 -18.702 42.252

CC 60.2965 20.389 2.957 0.004 20.014 100.579

==============================================================================

Omnibus: 3.725 Durbin-Watson: 1.881

Prob(Omnibus): 0.155 Jarque-Bera (JB): 2.846

Skew: -0.199 Prob(JB): 0.241

Kurtosis: 2.466 Cond. No. 18.6

==============================================================================

Independent variable: IA; dependent variable: FMWT_post_

OLS Regression Results

==============================================================================

Dep. Variable: fmt_M12 R-squared: 0.049

Model: OLS Adj. R-squared: 0.042

Method: Least Squares F-statistic: 7.772

Date: Tue, 26 Jul 2022 Prob (F-statistic): 0.00599

Time: 15:30:45 Log-Likelihood: -689.35

No. Observations: 154 AIC: 1383.

Df Residuals: 152 BIC: 1389.

Df Model: 1

Covariance Type: nonrobust

==============================================================================

coef std err t P>|t| [0.025 0.975]

------------------------------------------------------------------------------

const 26.3382 11.172 2.357 0.020 4.265 48.411

IA 0.0588 0.021 2.788 0.006 0.017 0.100

==============================================================================

Omnibus: 4.607 Durbin-Watson: 1.853

Prob(Omnibus): 0.100 Jarque-Bera (JB): 3.240

Skew: -0.203 Prob(JB): 0.198

Kurtosis: 2.417 Cond. No. 3.43e+03

==============================================================================

Independent variables: CC, age at implantation, duration of hearing impairment; dependent variable: FMWT_post_

OLS Regression Results

==============================================================================

Dep. Variable: fmt_M12 R-squared: 0.177

Model: OLS Adj. R-squared: 0.161

Method: Least Squares F-statistic: 10.76

Date: Tue, 26 Jul 2022 Prob (F-statistic): 1.90e-06

Time: 15:30:45 Log-Likelihood: -678.17

No. Observations: 154 AIC: 1364.

Df Residuals: 150 BIC: 1376.

Df Model: 3

Covariance Type: nonrobust

==========================================================================================

coef std err t P>|t| [0.025 0.975]

------------------------------------------------------------------------------------------

const 37.4814 16.445 2.279 0.024 4.988 69.975

CC 61.6375 19.149 3.219 0.002 23.800 99.475

implant_age -0.5006 0.116 -4.313 0.000 -0.730 -0.271

ipsi_impaired_duration 0.1957 0.106 1.853 0.066 -0.013 0.404

==============================================================================

Omnibus: 2.682 Durbin-Watson: 1.913

Prob(Omnibus): 0.262 Jarque-Bera (JB): 2.043

Skew: -0.121 Prob(JB): 0.360

Kurtosis: 2.491 Cond. No. 1.05e+03

==============================================================================

Independent variables: IA, age at implantation, duration of hearing impairment; dependent variable: FMWT_post_

OLS Regression Results

==============================================================================

Dep. Variable: fmt_M12 R-squared: 0.171

Model: OLS Adj. R-squared: 0.155

Method: Least Squares F-statistic: 10.34

Date: Tue, 26 Jul 2022 Prob (F-statistic): 3.14e-06

Time: 15:30:45 Log-Likelihood: -678.71

No. Observations: 154 AIC: 1365.

Df Residuals: 150 BIC: 1378.

Df Model: 3

Covariance Type: nonrobust

==========================================================================================

coef std err t P>|t| [0.025 0.975]

------------------------------------------------------------------------------------------

const 52.0056 13.158 3.952 0.000 26.006 78.005

IA 0.0603 0.020 3.043 0.003 0.021 0.099

implant_age -0.4984 0.116 -4.279 0.000 -0.729 -0.268

ipsi_impaired_duration 0.2004 0.106 1.890 0.061 -0.009 0.410

==============================================================================

Omnibus: 2.840 Durbin-Watson: 1.896

Prob(Omnibus): 0.242 Jarque-Bera (JB): 2.126

Skew: -0.124 Prob(JB): 0.345

Kurtosis: 2.481 Cond. No. 4.34e+03

==============================================================================

Independent variables: CC, age at implantation, duration of hearing impairment, FMWT_pre_;

dependent variable: FMWT_post_

OLS Regression Results

==============================================================================

Dep. Variable: fmt_M12 R-squared: 0.337

Model: OLS Adj. R-squared: 0.315

Method: Least Squares F-statistic: 15.24

Date: Tue, 26 Jul 2022 Prob (F-statistic): 4.17e-10

Time: 15:30:45 Log-Likelihood: -535.61

No. Observations: 125 AIC: 1081.

Df Residuals: 120 BIC: 1095.

Df Model: 4

Covariance Type: nonrobust

==========================================================================================

coef std err t P>|t| [0.025 0.975]

------------------------------------------------------------------------------------------

const 29.3497 16.200 1.812 0.073 -2.724 61.424

CC 77.4923 18.388 4.214 0.000 41.086 113.899

implant_age -0.6432 0.122 -5.260 0.000 -0.885 -0.401

ipsi_impaired_duration 0.1949 0.108 1.807 0.073 -0.019 0.409

db_opt_headphones 0.2720 0.071 3.841 0.000 0.132 0.412

==============================================================================

Omnibus: 1.966 Durbin-Watson: 1.927

Prob(Omnibus): 0.374 Jarque-Bera (JB): 1.699

Skew: -0.155 Prob(JB): 0.428

Kurtosis: 2.521 Cond. No. 1.09e+03

==============================================================================

Independent variables: IA, age at implantation, duration of hearing impairment, FMWT_pre_;

dependent variable: FMWT_post_

OLS Regression Results

==============================================================================

Dep. Variable: fmt_M12 R-squared: 0.326

Model: OLS Adj. R-squared: 0.303

Method: Least Squares F-statistic: 14.48

Date: Tue, 26 Jul 2022 Prob (F-statistic): 1.12e-09

Time: 15:30:45 Log-Likelihood: -536.67

No. Observations: 125 AIC: 1083.

Df Residuals: 120 BIC: 1097.

Df Model: 4

Covariance Type: nonrobust

==========================================================================================

coef std err t P>|t| [0.025 0.975]

------------------------------------------------------------------------------------------

const 48.9264 13.056 3.747 0.000 23.076 74.777

IA 0.0745 0.019 3.929 0.000 0.037 0.112

implant_age -0.6442 0.123 -5.223 0.000 -0.888 -0.400

ipsi_impaired_duration 0.1956 0.109 1.797 0.075 -0.020 0.411

db_opt_headphones 0.2555 0.071 3.590 0.000 0.115 0.396

==============================================================================

Omnibus: 2.269 Durbin-Watson: 1.917

Prob(Omnibus): 0.322 Jarque-Bera (JB): 1.740

Skew: -0.113 Prob(JB): 0.419

Kurtosis: 2.468 Cond. No. 4.28e+03

==============================================================================

Independent variable: CC; dependent variable: HSM

OLS Regression Results

==============================================================================

Dep. Variable: hsm_10_db_M12 R-squared: 0.006

Model: OLS Adj. R-squared: -0.001

Method: Least Squares F-statistic: 0.9116

Date: Tue, 26 Jul 2022 Prob (F-statistic): 0.341

Time: 15:30:45 Log-Likelihood: -669.93

No. Observations: 143 AIC: 1344.

Df Residuals: 141 BIC: 1350.

Df Model: 1

Covariance Type: nonrobust

==============================================================================

coef std err t P>|t| [0.025 0.975]

------------------------------------------------------------------------------

const 28.8527 19.353 1.491 0.138 -9.407 67.112

CC 24.3863 25.541 0.955 0.341 -26.106 74.878

==============================================================================

Omnibus: 30.946 Durbin-Watson: 1.928

Prob(Omnibus): 0.000 Jarque-Bera (JB): 7.075

Skew: -0.100 Prob(JB): 0.0291

Kurtosis: 1.929 Cond. No. 18.2

==============================================================================

Independent variable: IA; dependent variable: HSM

OLS Regression Results

==============================================================================

Dep. Variable: hsm_10_db_M12 R-squared: 0.008

Model: OLS Adj. R-squared: 0.001

Method: Least Squares F-statistic: 1.181

Date: Tue, 26 Jul 2022 Prob (F-statistic): 0.279

Time: 15:30:45 Log-Likelihood: -669.80

No. Observations: 143 AIC: 1344.

Df Residuals: 141 BIC: 1350.

Df Model: 1

Covariance Type: nonrobust

==============================================================================

coef std err t P>|t| [0.025 0.975]

------------------------------------------------------------------------------

const 32.2251 13.966 2.307 0.022 4.616 59.834

IA 0.0286 0.026 1.087 0.279 -0.023 0.081

==============================================================================

Omnibus: 31.596 Durbin-Watson: 1.928

Prob(Omnibus): 0.000 Jarque-Bera (JB): 7.132

Skew: -0.100 Prob(JB): 0.0283

Kurtosis: 1.924 Cond. No. 3.36e+03

==============================================================================

Independent variable: CC; dependent variable: ΔFMWT

OLS Regression Results

================================================================================

Dep. Variable: delta_M12_headphones R-squared: 0.083

Model: OLS Adj. R-squared: 0.075

Method: Least Squares F-statistic: 11.08

Date: Tue, 26 Jul 2022 Prob (F-statistic): 0.00115

Time: 15:30:45 Log-Likelihood: -581.79

No. Observations: 125 AIC: 1168.

Df Residuals: 123 BIC: 1173.

Df Model: 1

Covariance Type: nonrobust

==============================================================================

coef std err t P>|t| [0.025 0.975]

------------------------------------------------------------------------------

const -32.4429 19.545 -1.660 0.099 -71.131 6.246

CC 86.7434 26.063 3.328 0.001 35.154 138.333

==============================================================================

Omnibus: 4.324 Durbin-Watson: 1.755

Prob(Omnibus): 0.115 Jarque-Bera (JB): 4.320

Skew: -0.420 Prob(JB): 0.115

Kurtosis: 2.646 Cond. No. 17.7

==============================================================================

Independent variable: IA; dependent variable: ΔFMWT

OLS Regression Results

================================================================================

Dep. Variable: delta_M12_headphones R-squared: 0.057

Model: OLS Adj. R-squared: 0.049

Method: Least Squares F-statistic: 7.376

Date: Tue, 26 Jul 2022 Prob (F-statistic): 0.00756

Time: 15:30:45 Log-Likelihood: -583.54

No. Observations: 125 AIC: 1171.

Df Residuals: 123 BIC: 1177.

Df Model: 1

Covariance Type: nonrobust

==============================================================================

coef std err t P>|t| [0.025 0.975]

------------------------------------------------------------------------------

const -5.9710 14.231 -0.420 0.676 -34.141 22.199

IA 0.0736 0.027 2.716 0.008 0.020 0.127

==============================================================================

Omnibus: 3.738 Durbin-Watson: 1.725

Prob(Omnibus): 0.154 Jarque-Bera (JB): 3.766

Skew: -0.396 Prob(JB): 0.152

Kurtosis: 2.693 Cond. No. 3.22e+03

==============================================================================

Independent variables: CC, age at implantation, duration of hearing impairment, FMWT_pre_;

dependent variable: ΔFMWT

OLS Regression Results

================================================================================

Dep. Variable: delta_M12_headphones R-squared: 0.562

Model: OLS Adj. R-squared: 0.547

Method: Least Squares F-statistic: 38.46

Date: Tue, 26 Jul 2022 Prob (F-statistic): 1.10e-20

Time: 15:30:45 Log-Likelihood: -535.61

No. Observations: 125 AIC: 1081.

Df Residuals: 120 BIC: 1095.

Df Model: 4

Covariance Type: nonrobust

==========================================================================================

coef std err t P>|t| [0.025 0.975]

------------------------------------------------------------------------------------------

const 29.3497 16.200 1.812 0.073 -2.724 61.424

CC 77.4923 18.388 4.214 0.000 41.086 113.899

implant_age -0.6432 0.122 -5.260 0.000 -0.885 -0.401

ipsi_impaired_duration 0.1949 0.108 1.807 0.073 -0.019 0.409

db_opt_headphones -0.7280 0.071 -10.281 0.000 -0.868 -0.588

==============================================================================

Omnibus: 1.966 Durbin-Watson: 1.927

Prob(Omnibus): 0.374 Jarque-Bera (JB): 1.699

Skew: -0.155 Prob(JB): 0.428

Kurtosis: 2.521 Cond. No. 1.09e+03

==============================================================================

Independent variables: IA, age at implantation, duration of hearing impairment, FMWT_pre_;

dependent variable: ΔFMWT

OLS Regression Results

================================================================================

Dep. Variable: delta_M12_headphones R-squared: 0.554

Model: OLS Adj. R-squared: 0.539

Method: Least Squares F-statistic: 37.31

Date: Tue, 26 Jul 2022 Prob (F-statistic): 3.00e-20

Time: 15:30:45 Log-Likelihood: -536.67

No. Observations: 125 AIC: 1083.

Df Residuals: 120 BIC: 1097.

Df Model: 4

Covariance Type: nonrobust

==========================================================================================

coef std err t P>|t| [0.025 0.975]

------------------------------------------------------------------------------------------

const 48.9264 13.056 3.747 0.000 23.076 74.777

IA 0.0745 0.019 3.929 0.000 0.037 0.112

implant_age -0.6442 0.123 -5.223 0.000 -0.888 -0.400

ipsi_impaired_duration 0.1956 0.109 1.797 0.075 -0.020 0.411

db_opt_headphones -0.7445 0.071 -10.457 0.000 -0.885 -0.604

==============================================================================

Omnibus: 2.269 Durbin-Watson: 1.917

Prob(Omnibus): 0.322 Jarque-Bera (JB): 1.740

Skew: -0.113 Prob(JB): 0.419

Kurtosis: 2.468 Cond. No. 4.28e+03

==============================================================================
